# Whole anterior visual pathway segmentation from high-resolution MRI using artificial intelligence

**DOI:** 10.1186/s41747-026-00741-y

**Published:** 2026-06-05

**Authors:** Andrea Diociasi, Emanuele Pravatà, Luca Carmisciano, Oliver C. Kiersnowski, Iester Michele, Kevin Klein Gunnewiek, Luigi Lorenzini, Lorenzo Gualco, Matteo Pardini, Luca Roccatagliata, Chiara Zecca, Claudio Gobbi, Roberto Guidotti, Andrea Chincarini

**Affiliations:** 1 Department of Neuroscience, Rehabilitation, Ophthalmology, Genetics, Maternal and Child Health (DINOGMI), University of Genova, Genova, Italy; 2 Department of Internal Medicine (DIMI), University of Genova, Genova, Italy; 3 Department of Neuroscience, Imaging and Clinical Sciences, Centro I.T.A.B., Università degli Studi G. D’Annunzio di Chieti-Pescara, Chieti, Italy; 4 Neurocenter of Southern Switzerland, EOC, Lugano, Switzerland; 5 Department of Health Sciences, Section of Biostatistics, University of Genova, Genova, Italy; 6 IRCCS Ospedale Policlinico San Martino, Genova, Italy; 7 Princess Máxima Center for Pediatric Oncology, Utrecht, The Netherlands; 8 Amsterdam University Medical Centre, Vrije Universiteit, Amsterdam, The Netherlands; 9 Radiology Unit, Santi Antonio e Biagio e Cesare Arrigo University Hospital, Alessandria, Italy; 10 Faculty of Biomedical Sciences, Università della Svizzera Italiana, Lugano, Switzerland; 11 Istituto Nazionale di Fisica Nucleare, Genova, Italy

**Keywords:** Artificial intelligence, Deep learning, Magnetic resonance imaging, Multiple sclerosis, Visual pathways

## Abstract

**Objective:**

Manual segmentation of the whole anterior visual pathway (aVP) from high-resolution magnetic resonance imaging (MRI) is time-consuming and prone to inter-rater variability. We developed and validated a fully automated deep learning framework, “aVP-seg,” to perform rapid, multiclass segmentation of the optic nerves, chiasm, and optic tracts in healthy volunteers and multiple sclerosis (MS) patients.

**Materials and methods:**

We developed and validated a cascaded two-stage three-dimensional convolutional neural network (principal segmentation + refinement) for automated multiclass segmentation of the aVP from 0.6-mm isotropic three-dimensional constructive interference in steady state (CISS) MRI. The model was trained and evaluated in 34 healthy controls and 46 MS patients. Ground truth was derived from manual segmentations by two expert radiologists. Spatial agreement metrics included Dice similarity coefficient (DSC), 95th percentile Hausdorff distance (HD95), and volumetric similarity.

**Results:**

Agreement with the ground truth for the whole aVP was high (DSC 0.86 ± 0.03, mean ± standard deviation; 95% confidence interval (CI) 0.85–0.86). Boundary alignment was strong (HD95 1.18 mm ± 0.54; 95% CI 1.06–1.30) and volumetric similarity was high (0.96 ± 0.04; 95% CI 0.95–0.97). Accuracy was consistent for the left and right optic nerves (DSC 0.85–0.86 ± 0.05–0.04) and chiasm (DSC 0.83 ± 0.09), but lower for the left and right optic tracts (DSC 0.74–0.75 ± 0.07–0.07).

**Conclusion:**

The aVP-seg provided accurate, automated multiclass segmentation of the whole aVP from high-resolution CISS MRI. This tool may standardize and accelerate the extraction of quantitative biomarkers of aVP integrity in neuro-ophthalmic conditions.

**Relevance statement:**

Automated multiclass segmentation of the entire anterior visual pathway enables standardized and reproducible preparation of MRI data for quantitative analysis. This approach facilitates future assessment of optic pathway involvement in MS and other neuro-ophthalmic disorders.

**Key Points:**

- aVP-seg enabled fully automated segmentation of the entire anterior visual pathway from high-resolution CISS MRI data.
- Automated segmentation reduces processing time and operator-dependent variability.
- aVP-seg shows robust performance across both healthy subjects and MS patients.

**Graphical Abstract:**

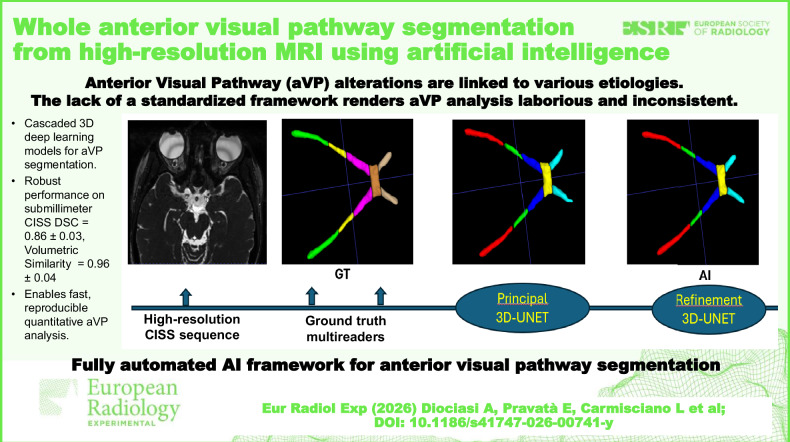

## Background

The anterior visual pathway (aVP) may be targeted by a variety of neuro-ophthalmic conditions [1]. In particular, multiple sclerosis (MS) is a well-established cause of aVP involvement, in which both focal inflammatory lesions and diffuse neurodegenerative processes may contribute to structural damage along the entire pathway [2–5]. Characterizing aVP damage is therefore relevant for quantitative assessment and longitudinal monitoring of neurodegenerative and neuroreparative processes in MS.

While optical coherence tomography is an established method to provide indirect estimation of the aVP integrity from the retinal nerve fiber layer [6], Three-dimensional (3D) high-resolution magnetic resonance imaging (MRI) offers a complementary perspective by enabling anatomical assessment of the aVP along its entire intracranial and intraorbital course, beyond the retina. To this end, atlas-based frameworks and validated anatomical landmarks provide a robust reference for population-level aVP biometry [7], but their application at the individual-subject level may be challenged by inter-individual anatomical variability and disease-related structural alterations. In addition, manual extraction of the aVP structure from non-CNS tissues in multiple patients requires time-consuming segmentations that are prone to operator-dependent variability and reproducibility issues, as previously demonstrated [8]. To overcome these limitations, we developed an AI-driven model (the “aVP-seg”) based on a 3D V-Net architecture, for the automatic extraction of the entire aVP from 0.6-mm^3^ isotropic resolution 3D-constructive interference in steady state (CISS) images, acquired in both healthy participants and participants with MS. The model was trained and tested against ground-truth (GT) segmentations derived from the union of independent manual segmentations performed by two readers, using previously defined anatomical landmarks [7].

## Methods

### Study design and ethics

This was a prospective, single-center study conducted at the Neurocenter of Southern Switzerland. The study was approved by the local Ethics Committee of Canton Ticino, Switzerland (Ref. 2017-00814; CE3224), and written informed consent was obtained from all participants.

### Participants

Thirty-four healthy controls and forty-eight patients with relapsing-remitting MS were consecutively recruited at the Neurocenter of Southern Switzerland between June 2017 and June 2019. The exclusion criteria were spherical refractive errors worse than -6 D, history of brain tumors, glaucoma, severe eye and/or head trauma, neurological or ophthalmological diseases other than MS, and clinical relapses within 3 months of enrollment. Two patients were excluded because of motion-corrupted images, resulting in 46 patients included in the final analysis, for a total of 80 participants. Participants’ demographic characteristics are summarized in Table 1.

**Table 1:** Demographic and clinical characteristics of participants included in the study

Characteristic	Healthy controls	MS patients	*p*-value
Number	34	46	–
Mean age (standard deviation)	36.8 (8.2)	38.3 (10.3)	0.478^a^
Gender (F/M)	18/16	31/15	0.190^b^
Mean disease duration in months (standard deviation)	-	102 (88.5)	
Median EDSS (interquartile range)	-	2 (1–3)	

*EDSS* Expanded disability status scale, *MS* Multiple sclerosis

^a^ Independent *t*-test

^b^ χ^2^ test

### Data acquisition and preparation

Images were acquired on a 3-T MRI “Skyra” scanner (Siemens), using a 64-channel head coil. To prevent degradation of image quality due to the subject’s eye movements, participants were instructed to remain still with their eyes closed and maintain a fixed gaze [9]. Constructive interference in the steady state (CISS) images acquired on the orbits and intracranial aVP were preferred to current standard 3D techniques employed for the whole brain (*i.e*., T1-weighted magnetization‑prepared rapid gradient-echo or inversion-recovery fast spoiled gradient-echo), for the superior signal contrast at the interface between the aVP and surrounding cerebrospinal fluid, and for the ability to obtain sub-millimeter spatial resolution images in a relatively short time [7].

Furthermore, compared with both T2- and T1-weighted images, CISS sequences exhibit intrinsically low sensitivity to lesions, thereby minimizing the risk of tissue misclassification related to the reduced contrast between the nerve tissue and the cerebrospinal fluid potentially occurring with T2-hyperintense and T1-hypointense lesions [10]. The following scan parameters were used: repetition time = 8.1 ms; echo time = 3.74 ms; field of view = 150 mm^2^; matrix = 245 × 245; acquired voxel size = 0.6 × 0.6 × 0.6 mm^3^; number of slices = 72; scan duration = 3:11 min:s. All images were visually quality checked for artifacts by an experienced neuroradiologist (E.P.) and subsequently cropped to a standardized matrix of 256 × 256 × 64 voxels.

### Generation of the ground truth

To ensure anatomical consistency, the aVP was independently segmented on the CISS images by two board-certified radiologists (A.D., L.C., R1 and R2, respectively, each with more than 5 years of experience in central nervous system anatomy segmentation, including the aVP), into binary labels, according to previously defined anatomical landmarks [7]. The images were presented in a randomized order using ITK-SNAP v3.6.0. Separate labels were assigned to each aVP segment, *i.e*., the left and right optic nerve (ONL and ONR, respectively) and its intraorbital (iOrb), intracanalicular (iCan) and intracranial (iCran) subdivisions, the optic chiasm (ONC), and left and right optic tracts (OTL and OTR, respectively) [7].

To establish a reference standard (ground truth) for model training, the segmentations from R1 and R2 were combined (R1 ∪ R2) using a union-based approach, in which any voxel delineated by either reader was included. This method was preferred over a geometric or probabilistic averaging strategy, as it ensured inclusion of all potential aVP regions identified by either radiologist, thereby providing a more comprehensive representation of aVP morphology and size, while accounting for possible inter-reader variability in anatomical landmark identification. The inter-reader agreement analysis is described in detail in the Supplementary material.

### AI model architecture and training

**Fig. 1:**
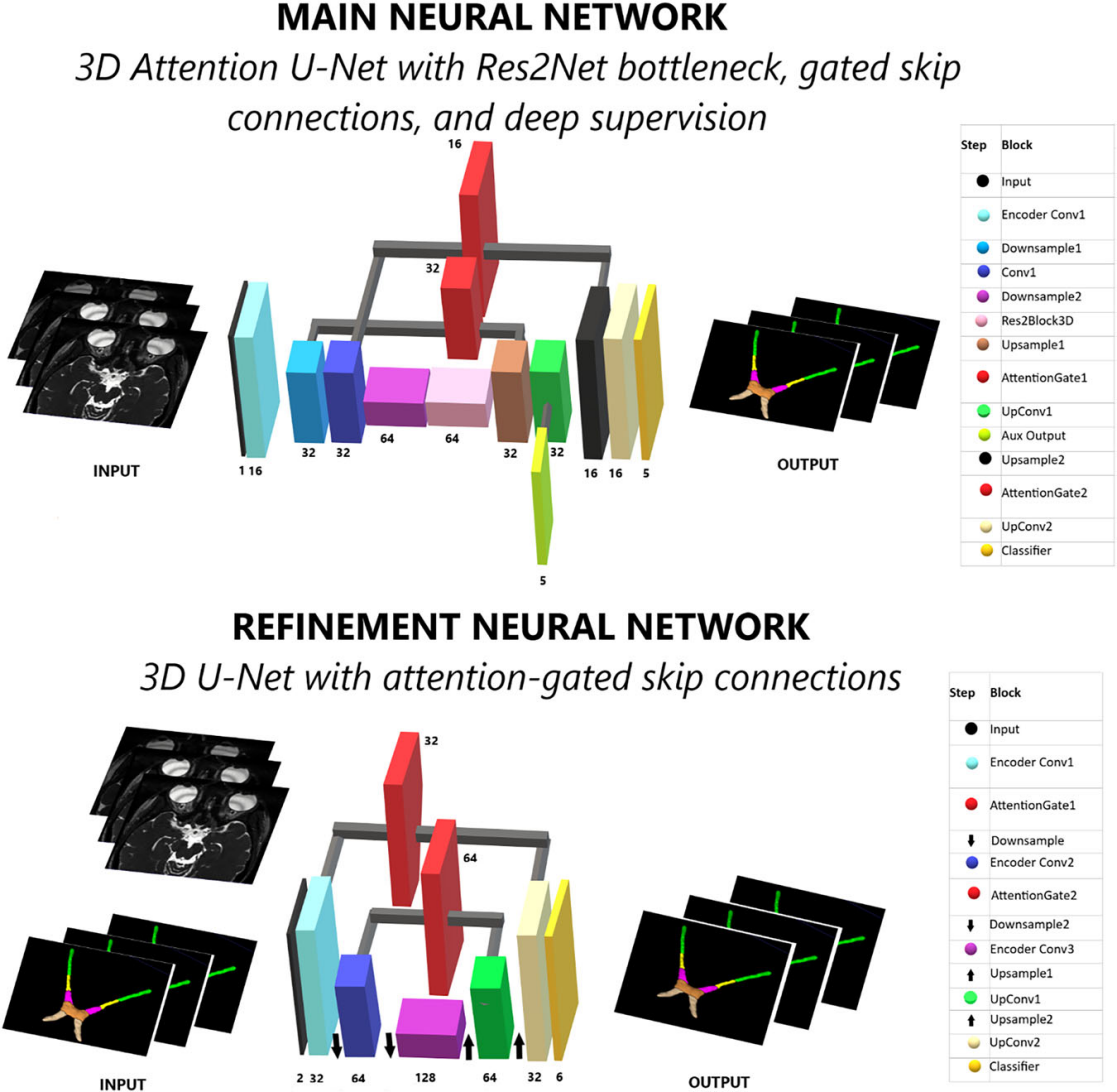
Overview of the two-stage segmentation aVP-seg framework. (Top) Main neural network: a 3D Attention U-Net with a Res2Net bottleneck, attention-gated skip connections, and deep supervision. The model uses the CISS MRI volume as an input and produces an initial multiclass segmentation. (Bottom) Refinement neural network: A cascaded 3D U-Net with attention-gated skip connections. This network takes as input both the CISS MRI and the initial segmentation predicted by the main network, and outputs a refined segmentation with improved anatomical consistency and boundary accuracy. 3D, Three-dimensional; aVP, Anterior visual pathway; CISS, Constructive interference in steady state; MRI, Magnetic resonance imaging

The primary segmentation model was a custom 3D encoder-decoder architecture inspired by V-Net. The network featured strided convolutional downsampling, a symmetric upsampling decoder path, Res2Block bottlenecks, attention-gated skip connections, and deep supervision through an auxiliary output head. Unlike canonical V-Net, residual units were limited to the bottleneck, and ReLU activations were used throughout (Fig. 1, upper part). Model evaluation was performed using Monte Carlo cross-validation with an 80/20 train–validation split. The random partitioning was repeated across multiple runs until each subject had been included in the validation set at least once. Full training details are provided in the Supplementary material. In contrast to most previously reported methods, which primarily generate a single aVP mask, the proposed framework performs fully automated multiclass segmentation, producing nine anatomically distinct labels including the ONL and ONR (each subdivided into iOrb, iCan and iCran segments), ONC, OTL and OTR. A selection of previously reported automated approaches for aVP segmentation is summarized in Table 2 for contextual comparison.

**Table 2:** Comparison of anterior visual pathway (aVP) segmentation methods reported in the literature and the proposed aVP-seg framework

Method	Year	*N*	MRI technique	Voxel size (mm³)	DSC	HD95 (mm)	ASSD (mm)	VS	Output	Population
PAScAL (Mansoor et al)	2016	40	T1w	0.4 × 0.4 × 0.6	0.779	–	–	–	aVP (probability map)	Pediatric patients (pathology not specified)
3D FCN + SPDM (Zhao et al)	2019	93	T1w	Patients 1 × 1 × 1	0.85 ± 0.01	4.64 ± 1.41	0.37 ± 0.04	–	aVP Single class	(pathology not specified)
3D U-Net (Ai et al)	2020	93	T1w	1 × 1 × 1	0.86 ± 0.01	3.56 ± 1.89	0.34 ± 0.05	–	aVP Single class	Healthy controls
TPSN (Li et al)	2021	102	T1w + DTI	1.25 × 1.25 × 1.25	0.85 ± 0.02	2.33	0.16	–	aVP Single class	Healthy controls
3D U-Net (van Elst et al)	2022	40	T2w	0.25 × 0.25 × 0.7	0.84	0.64	0.14	–	ON Single class	Pediatric patients (optic pathway glioma)
CNTSeg (Xie et al)	2023	102	T1w + DTI	1.25 × 1.25 × 1.25	0.82 ± 0.02	–	–	–	aVP Single class	Healthy controls
3D UX-Net (Han et al)	2025	119	T1w	0.90 × 0.90 × 1.0	0.893 ± 0.017	1.00 [1]	0.234 [0.188–0.273]	–	aVP Single class	Patients without orbital pathology
aVP-seg (this study)	2025	80	CISS	0.6 × 0.6 × 0.6	0.86 ± 0.03	1.18 ± 0.54	0.41 ± 0.15	0.96	aVP Multiclass (9 Labels)	Healthy controls + MS patients

Values are reported as mean ± standard deviation or median [interquartile range], as provided by the original studies

*aVP* Anterior visual pathway, *ASSD* Average symmetric surface distance, *CISS* Constructive interference in steady state, *DSC* Dice similarity coefficient, *DTI* Diffusion tensor imaging, *fODF* Fiber orientation distribution function, *HD95* 95th percentile Hausdorff distance, *MRI* Magnetic resonance imaging, *SPDM* Spatial probabilistic distribution map, *T1w* T1-weighted, *T2w* T2-weighted, *TPSN* Two-parallel-stage network, *VS* Volumetric similarity

### Segmentation refinement

To enhance prediction accuracy, especially at anatomical boundaries, we implemented a second-stage refinement model based on a lightweight 3D U-Net (Fig. 1, lower part). This model took as input both the CISS image and the corresponding segmentation output from the principal network. It used attention gates and a boundary-weighted Dice loss, where voxels near structure boundaries received a higher loss penalty. The refinement model was trained per fold using predictions from the principal network with a mean Dice similarity coefficient (DSC) ≥ 0.7, ensuring that only sufficiently accurate segmentations were used to guide refinement. The predictions of the multiple validation models were combined using a majority voting threshold of 0.5. To further ensure anatomical consistency, post-processing procedures were automatically applied, including cluster removal, side-aware label enforcement, gap interpolation, and continuity correction across the optic nerve–chiasm–tract transitions. Full details of the entire pipeline are provided in the Supplementary material.

### Spatial similarity metrics

The DSC was calculated for each subject to quantify the overlap between the predicted segmentation and the reference standard. DSC was defined as

$${DSC}(A,\,B)=2{|A}\cap {B|}/({|A|}+{|B|})$$

where ${{{\rm{| }}}}A{{{\rm{| }}}}$ represents the volume of the test segmentation, ${{{\rm{| }}}}B{{{\rm{| }}}}$ the reference segmentation, and ${{{\rm{| }}}}A\cap B{{{\rm{| }}}}$ their intersection. The mean Hausdorff distance (HD) was defined as the maximum distance between any point in one segmentation and the closest point in the other, capturing the worst-case error in boundary alignment. It was calculated as H(A, B) = max(supₐ∈A infᵦ∈B d(a, b), supᵦ∈B infₐ∈A d(b, a)), where d(a, b) represents the Euclidean distance between a point in A and its nearest neighbor in B, and vice versa [11]. The 95th percentile Hausdorff distance (HD95) and the average symmetric surface distance were also computed to reduce sensitivity to outliers. Voxel-wise classification performance was further quantified using precision and recall. Volumetric similarity (VS) was defined as

$${VS}(A,B)=1-({||A|}-{|B||}/({|A|}+{|B|})).$$

### Statistics

Continuous variables were summarized as mean ± standard deviation (SD) or median with interquartile range (IQR). Categorical variables were summarized as frequencies and percentages. For demographic variables (Table 1), group differences between healthy controls and participants with MS were assessed using the Student’s *t*-test for continuous variables, and the chi-square test for categorical variables.

For all spatial similarity metrics, the mean and corresponding 95% confidence interval (CI) were calculated assuming a t-distribution with *N* - 1 degrees of freedom. All statistical analyses were conducted using R software (version 4.3.0; R Foundation for Statistical Computing).

## Results

### Ground-truth consistency

Inter-reader agreement for the ground-truth (GT) masks was high. The mean DSC between the radiologists (R1 and R2) was 0.83 (SD ± 0.03; 95% CI 0.82–0.84), with a corresponding mean Jaccard index of 0.71 (SD ± 0.05; 95% CI 0.70–0.72). Precision and recall were respectively 0.85 (SD ± 0.05; 95% CI 0.84–0.86) and 0.82 (SD ± 0.05; 95% CI 0.81–0.83).

Boundary similarity metrics further confirmed the robustness of the GT. The HD95 showed a low mean of 1.09 mm (SD ± 0.25; 95% CI 1.03–1.14), while the HD averaged 5.94 mm (SD ± 2.77; 95% CI 5.33–6.56). Average symmetric surface distance remained small (mean 0.44 mm, SD ± 0.10; 95% CI 0.42–0.46). See Supplementary Table S1.

Altogether, these results demonstrate that the manual GT masks exhibit high volumetric and boundary reproducibility, confirming their suitability as a reliable reference standard for benchmarking AI segmentation.

### aVP-seg spatial similarity

AI achieved a mean DSC of 0.86 (SD ± 0.03; 95% CI 0.85–0.86) and a mean Jaccard index of 0.75 (SD ± 0.05; 95% CI 0.74–0.76) against GT. Mean boundary similarity was 1.18 (expressed as HD95 - SD ± 0.54; 95% CI 1.06–1.30), 4.82 (expressed as HD - SD ± 2.49; 95% CI 4.27–5.37), and 0.41 (expressed as Average symmetric surface distance - SD ± 0.15; 95% CI 0.37–0.44). The VS was 0.96 (SD ± 0.04; 95% CI 0.95–0.97), while precision and recall were 0.87 (SD ± 0.05; 95% CI 0.86–0.88) and 0.85 (SD ± 0.06; 95% CI 0.84–0.86), respectively. Altogether, these results indicate very good overall agreement between AI and GT, in terms of segmentation boundary similarity. Table 3 presents a summary of the similarity results for the whole aVP.

**Table 3:** Summary of all spatial similarity metrics between the whole aVP artificial intelligence model and ground truth

Mean		95% Confidence interval		
		Lower	Upper	SD
DSC	0.86	0.85	0.86	0.03
Hausdorff distance	4.82	4.27	5.37	2.49
HD95	1.18	1.06	1.30	0.54
Jaccard	0.75	0.74	0.76	0.05
Precision	0.87	0.86	0.88	0.05
Recall	0.85	0.84	0.86	0.06
Volumetric similarity	0.96	0.95	0.97	0.04
ASSD	0.41	0.37	0.44	0.15

The 95% confidence interval of the mean assumes sample means follow a *t*-distribution with *N* minus 1 degrees of freedom. Higher values for DSC, Jaccard, Precision, Recall, and Volumetric similarity, and lower values for Hausdorff, HD95, and ASSD, indicate higher morphology similarity. Hausdorff distance, 95th percentile Hausdorff distance (HD95), and average symmetric surface distance (ASSD) are measured in mm. Volume-based metrics are expressed in mm³

The corresponding optic nerve, optic chiasm and Optic tract segment results are reported in the Supplementary Table S2

*SD* Standard deviation

DSC values were relatively higher for the ON (ONR = 0.86 ± 0.004; ONL = 0.85 ± 0.05) and for the ONC (0.83 ± 0.08), than for the OT (OTR = 0.75 ± 0.07; OTL = 0.74 ± 0.07), suggesting some limitation of the agreement between AI and GT at the level of the confluence between the aVP and the brain. As for the ON anatomical subdivisions, a mean DSC of 0.86 (SD ± 0.08; 95% CI 0.84–0.87) for iOrb, 0.71 (SD ± 0.09; 95% CI 0.69–0.73) for iCan, and 0.81 (SD ± 0.05; 95% CI 0.80–0.82) for iCran segment were achieved, suggesting relatively weaker spatial agreement at the level of the iCan subdivision. When interpreted in the context of the observed inter-reader agreement, the agreement achieved by aVP-seg falls within the range of variability between expert manual segmentations across overlap- and boundary-based metrics (DSC and HD95), indicating performance consistent with expert delineation variability. More detailed descriptions of each aVP segment and of each ON anatomical subdivision, including a summary of all similarity metrics, are provided in the Supplementary Table S2, and Supplementary paragraph “ON anatomical subdivisions.”

**Fig. 2:**
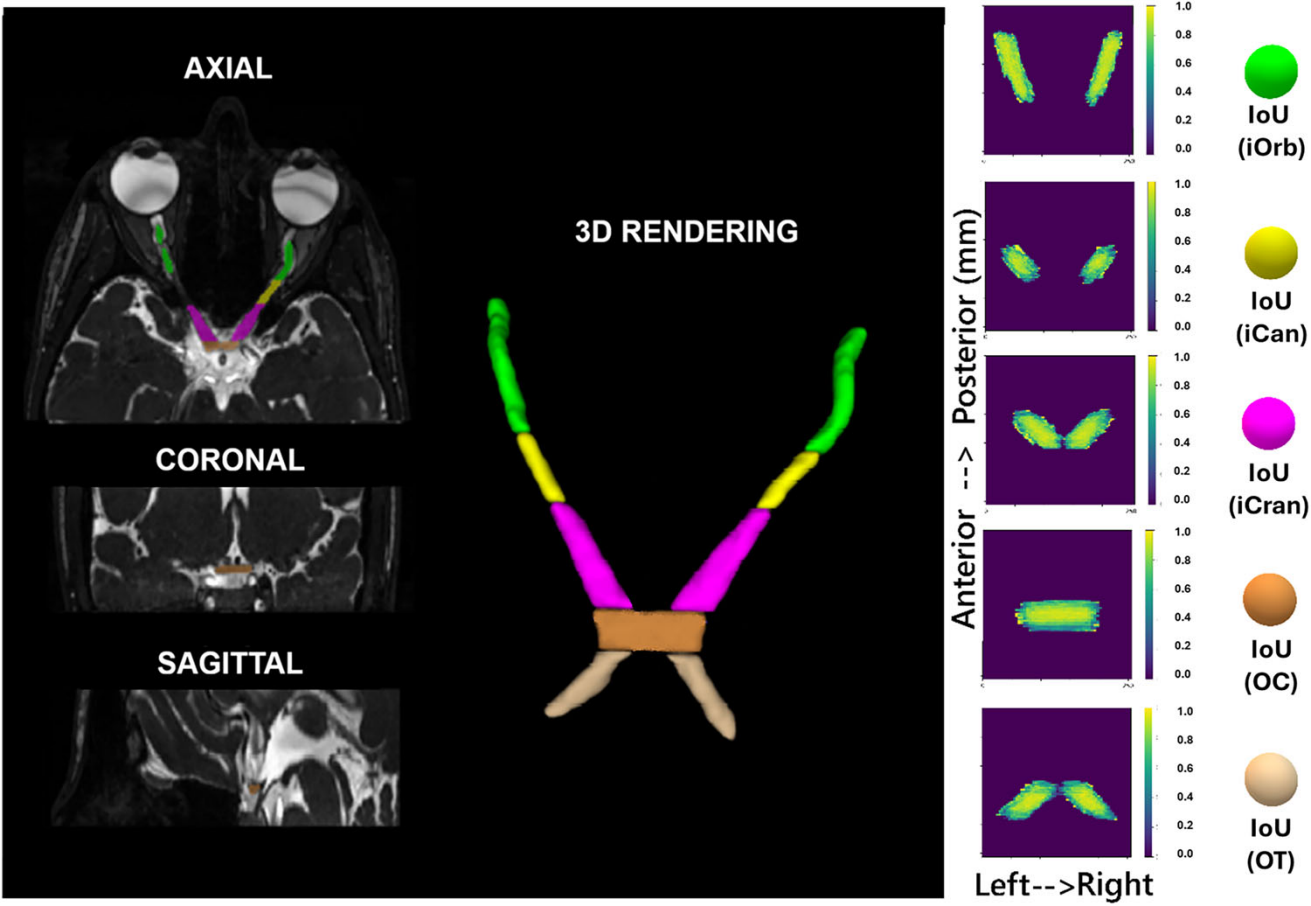
Multiplanar visualization and regional agreement of the optic-pathway segmentation. Left panel: Exemplary case showing axial, coronal, and sagittal plane CISS image reconstructions with the aVP segmentation highlighted in colors, according to each anatomical iOrb (green), iCan (yellow), iCran (magenta), OC (orange), OT (beige) subdivisions, and corresponding 3D-volume rendering. Right panel: Regional agreement maps per each anatomical subdivision, showing per-column intersection-over-union (IoU), also known as Jaccard Index, between prediction and ground truth, computed along the axial depth and displayed as a union-over-z projection (scale 0–1; brighter = better overlap; blank = no signal). Axes in mm (left ↔ right; anterior → posterior). aVP, Anterior visual pathway; CISS, Constructive interference in steady state; iCan, Intracanalicular optic nerve segment; iCran, Intracranial optic nerve segment; iOrb, Intraorbital optic nerve segment

**Fig. 3:**
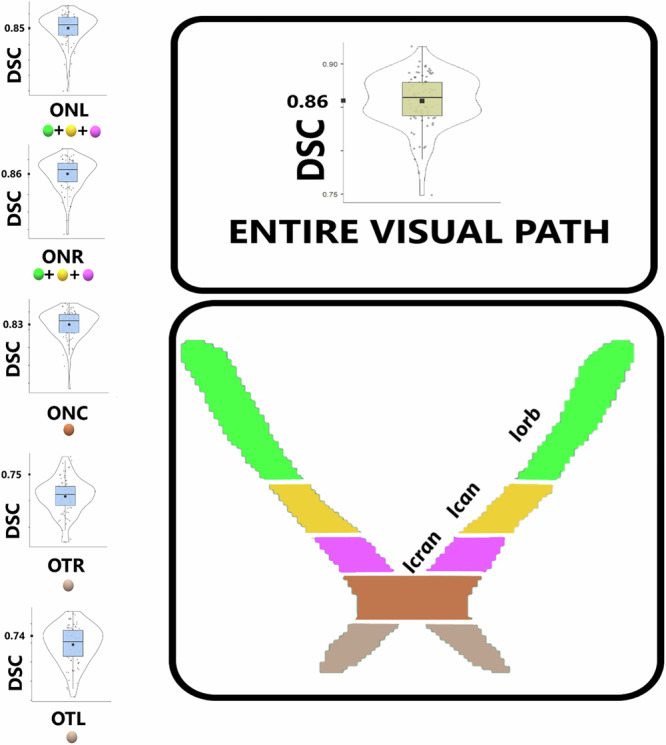
Regional and side-specific segmentation Dice similarity coefficient (DSC). A schematic representation of the aVP and its anatomical segments (iOrb = green, iCan = yellow, iCran = magenta, ONC = orange, OTR/OTL = beige). ONR and ONL DSC represent the left and right side averages. Violin plots show DSC distribution across subjects for aggregated structures: Entire Visual Path. ONR/ONL, Right/left optic nerve; ONC, Optic chiasm; OTR/OTL, Right/left optic tract. In each violin, the box marks the interquartile range, the horizontal line the median, the black marker the mean, and the gray dots individual subjects. DSC ranges 0–1 (higher is better)

Figure 2 presents a representative case of aVP automatic segmentation performed by AI, while Fig. 3 presents the DSC values distribution across subjects per each anatomical subdivision.

## Discussion

This work introduces a fully automated, two-stage deep learning framework for multiclass segmentation of the aVP from dedicated, high-resolution 3D-CISS MRI of the orbits, the “aVP-seg.” To our knowledge, this is the first model combining: (1) end-to-end multiclass predictions for the optic nerves, chiasm, and tracts based on sub-millimeter (0.6 mm isotropic) images, (2) a cascaded pair of distinct models (principal + refinement), and (3) suitability for patients with MS. aVP-seg is designed to improve diagnostic accuracy by mitigating operator-related bias, while cutting the time and manual effort required for data analysis preparation. Based on our institutional experience during the ground-truth (GT) generation, manual segmentation of the entire aVP on CISS images required approximately 30–45 min for a trained radiologist. In contrast, aVP-seg performs the same task in less than 60 s using a standard GPU.

Previously published MRI-based segmentation models for the aVP employed various architectures, tissue contrast, and spatial resolutions. Van Elst et al [12] applied a 3D U-Net model on high-resolution T2-weighted images in a cohort of pediatric patients with retinoblastoma. However, the resulting segmentation output was limited to the optic nerve. Ai et al [13, 14] and Li et al [15] applied T1-weighted and diffusion-informed networks, while Xie et al [16] combined diffusion and fiber-orientation data to enhance boundary definition, albeit additional manual editing steps were still required. Recently, Han et al [14] achieved a strong overall agreement with operator segmentations by using a 3D UX-Net architecture, but this study was limited to healthy participants. Earlier shape- or atlas-based and machine learning approaches [17–21] provided valuable anatomical priors through multi-atlas strategies, but lacked the capability for anatomical sub-segmentation, as well as effective solutions to minimize tissue misclassification, atlas misregistration, and imprecise nerve boundary delineation. Against this background, our approach introduces several advancements. First, it leverages the superior spatial and contrast resolution of the CISS sequence to optimize delineation of the aVP relative to its surrounding anatomical structures. Second, compared with conventional T1- and T2-weighted imaging, CISS images are relatively insensitive to contrast degradation caused by lesions, thereby minimizing tissue misclassification bias in diseased populations [7]. Furthermore, because the segmentation task relies on the nerve–cerebral spinal fluid interface rather than the lesion signal, MS pathology does not introduce a relevant domain shift for the proposed method. Third, aVP-seg provides a multiclass segmentation capability trained on an existing anatomical labeling framework, thereby enabling automated data preparation for region-wise biometry extraction and group-level comparisons.

Importantly, our model performed similarly to experienced radiologists in capturing subject-specific anatomical features in both healthy participants and patients with MS. This capability is crucial for the downstream processing of MRI data, enabling reliable extraction of aVP biomarkers at the individual-subject level, both in healthy and diseased populations. Indeed, evaluation of the aVP structure and monitoring of its changes can substantially improve the clinical management of people with MS. The growing interest in studying the optic nerve in MS is reflected by its incorporation into the most recent revision of the MS diagnostic criteria [22]. AI-based tools have the potential to advance the analysis of the optic nerve in MS. For instance, a 3D convolutional neural network has been developed to detect lesions within the optic nerve in people with MS [23]. However, there remains a lack of AI applications specifically designed for segmentation of the entire aVP and for the detection of optic nerve atrophy, a pathological process that, in addition to focal lesions, may affect the optic nerve in MS [20].

Although evaluated here in healthy participants and patients with MS, aVP-seg may be in principle applicable to other neurological or neuro-ophthalmological conditions related to aVP injury and/or degeneration, including neuromyelitis optica spectrum disorder and myelin oligodendrocyte glycoprotein antibody–associated disease [3], ischemic optic neuropathy, compressive or neoplastic/infiltrative lesions, hereditary and toxic–metabolic neuropathies, and trauma [2]. Application in such settings will, however, require disease-specific validation.

Our study is not without limitations. First, all data were acquired at a single center using a single scanner and a single CISS acquisition protocol. As a result, the generalizability of aVP-seg across different vendors, field strengths, coils, and acquisition settings cannot be directly assessed. Domain shift across scanners and protocols is a known challenge for deep learning–based medical image segmentation, and external multicenter validation is required to confirm robustness in heterogeneous clinical environments. Although standardized acquisition, expert quality control, and consistent preprocessing were used to reduce scanner-specific bias, these measures cannot replace independent external testing.

Future work will therefore focus on validation in multicenter, multi-vendor cohorts and on the evaluation of harmonization or domain-adaptation strategies to improve cross-scanner robustness. The absence of a quantitative external validation cohort represents another limitation. To provide an illustrative example of feasibility beyond the training domain, aVP-seg was additionally applied to a single external subject acquired at a different center with a different scanner but the same protocol (Supplementary Fig. S2); however, this qualitative example does not replace formal external validation. A formal sample size or power analysis was not performed, as the primary objective of this study was methodological validation of segmentation performance rather than hypothesis testing against a predefined clinical endpoint. The use of a union-based ground truth may result in mild volume overestimation due to the inclusion of reader-specific false positives. Mathematically, the union-based GT maximizes the denominator (|A| + |B|) in the DSC formula. If the model learns to predict the anatomically consistent ‘core’ (similar to the intersection or STAPLE), the Union GT treats the inter-reader disagreements as ‘missed’ tissue (False Negatives), thereby systematically lowering the DSC compared to an Intersection-based or STAPLE GT. Thus, our reported performance is a conservative lower bound.

In line with previous findings in the literature [19], relatively larger spatial discrepancies between AI- and reader-derived segmentations were observed at the level of the OT. These differences were primarily driven by tissue labeling failures at the point of confluence with the brain—*i.e*., end-slice truncation—rather than by core overlap, indicating boundary localization (Supplementary Fig. S1, right panel). Furthermore, the iCan segment also exhibited comparatively lower spatial consistency relative to the GT. We hypothesize that image degradation due to magnetic field inhomogeneity at the level of the bony optic canal may partially account for this effect (Supplementary Fig. S1, left panel). Another limitation of this study lies in the relatively small sample size, underscoring the need for validation in larger, multicenter cohorts to ensure model generalizability.

In conclusion, by providing consistent, multilabel automated segmentations, aVP-seg can substantially facilitate the extraction of biomarkers from high-resolution MRI of the entire aVP in both healthy individuals and patients with MS, thereby facilitating standardization and reproducibility.

## Supplementary information

**Additional file 1: Table S1.** Summary of all spatial similarity metrics between Reader 1 (R1) and Reader 2 (R2) for the merged aVP ground-truth masks. **Table S2.** Summary of all spatial similarity metrics between A.I. and GT, each aVP ON, OC and OT segment. Higher values for Dice, Jaccard, Precision, Recall, and Volumetric similarity, and lower values for Hausdorff, HD95, and Average surface distance, indicate higher morphology similarity. **Figure S1. Common anatomical pitfalls in the segmentation of the aVP. Left:** Intracanalicular segment CISS images (coronal, axial, sagittal) showing reduced contrast between the optic nerve and surrounding cerebrospinal fluid, leading to boundary uncertainty. **Right:** Optic tracts example from subject 0035 illustrating high inter-reader variability in defining the posterior truncation of the tract. Yellow ellipses indicate regions of disagreement between readers. **Figure S2.** Representative axial, coronal, and sagittal CISS images from an external subject acquired at a different center using a different scanner with the same acquisition protocol are shown, with the automated aVP-seg output overlaid. The segmentation delineates the optic nerves, chiasm, and optic tracts using color coding, together with the corresponding 3D volume rendering. This example is provided for illustrative purposes only; no quantitative evaluation was performed, and no conclusions regarding cross-scanner or cross-protocol generalizability can be drawn from a single case.

## Data Availability

Anonymized derived data and/or access to the segmentation framework may be made available from the corresponding author upon reasonable and legitimate request, subject to institutional approvals and data-sharing agreements.

## Abbreviations

3D: Three-dimensional
AI: Artificial intelligence
aVP: Anterior visual pathway
CI: Confidence interval
CISS: Constructive interference in steady state
DSC: Dice similarity coefficient
GT: Ground truth
HD: Hausdorff distance
HD95: 95th percentile Hausdorff distance
iCan: Intracanalicular optic nerve segment
iCran: Intracranial optic nerve segment
iOrb: Intraorbital optic nerve segment
MRI: Magnetic resonance imaging
MS: Multiple sclerosis
ON: Optic nerve
ONC: Optic chiasm
ONL: Left optic nerve
ONR: Right optic nerve
OT: Optic tract
OTL: Left optic tract
OTR: Right optic tract
SD: Standard deviation
VS: Volumetric similarity
