# Supplementary material for: Whole anterior visual pathway segmentation from high-resolution MRI using artificial intelligence
Source: Eur Radiol Exp. 2026 Jun 5;10:82. doi: 10.1186/s41747-026-00741-y (PMC13241553; doi:10.1186/s41747-026-00741-y)
Supplement: Supplementary file 1 — Additional file 1: Table S1. Summary of all spatial similarity metrics between Reader 1 (R1) and Reader 2 (R2) for the merged aVP ground-truth masks. Table S2. Summary of all spatial similarity metrics between A.I. and GT, each aVP ON, OC and OT segment. Higher values for Dice, Jaccard, Precision, Recall, and Volumetric similarity, and lower values for Hausdorff, HD95, and Average surface distance, indicate higher morphology similarity. Figure S1. Common anatomical pitfalls in the segmentation of the aVP. Left: Intracanalicular segment CISS images (coronal, axial, sagittal) showing reduced contrast between the optic nerve and surrounding cerebrospinal fluid, leading to boundary uncertainty. Right: Optic tracts example from subject 0035 illustrating high inter-reader variability in defining the posterior truncation of the tract. Yellow ellipses indicate regions of disagreement between readers. Figure S2. Representative axial, coronal, and sagittal CISS images from an external subject acquired at a different center using a different scanner with the same acquisition protocol are shown, with the automated aVP-seg output overlaid. The segmentation delineates the optic nerves, chiasm, and optic tracts using color coding, together with the corresponding 3D volume rendering. This example is provided for illustrative purposes only; no quantitative evaluation was performed, and no conclusions regarding cross-scanner or cross-protocol generalizability can be drawn from a single case. [file 41747_2026_741_MOESM1_ESM.pdf]

Whole anterior visual pathway segmentation from high-resolution MRI using artificial intelligence

ELECTRONIC SUPPLEMENTARY MATERIAL

Neural Network

We implemented a two-stage deep learning framework for automatic segmentation of the aVP from high-resolution 3D-CISS MRI, comprising a principal segmentation model and a subsequent refinement model. The principal model was based on a custom 3D encoder-decoder network inspired by V-Net, with strided convolution-based downsampling, Res2Block bottlenecks, attention-gated skip connections, and deep supervision. The input to the network consisted of normalized 3D-CISS images, rescaled to zero mean and unit variance, with image intensities constrained between 0 and 1. Ground truth segmentations were constructed by merging expert-drawn masks from two neuroradiologists using a union-based approach (R1 ∪ R2), in which any voxel labeled by at least one reader was included in the reference mask. Label values in the input masks were remapped such that optic nerves (left/right), optic chiasm, and optic tracts were relabeled to classes 1 through 5. The network’s encoder comprised two convolutional layers with batch normalization and ReLU activations, followed by two downsampling steps, each halving the spatial resolution and doubling the feature channels. The bottleneck incorporated a Res2Block3D unit with a scale of four, allowing internal feature grouping and split-fusion operations. The decoder included symmetric upsampling steps via transpose convolutions and incorporated spatial attention gates to conditionally reweight encoder features based on decoder activations. Skip connections were used throughout. An auxiliary output branch (deep supervision) was added after the first decoder stage and

upsampled to match the original resolution. The model returned both a main output and auxiliary segmentation map, both used for training. The loss function was a weighted combination of Dice loss (λ=0.6), cross-entropy loss (λ=0.3), and boundary-aware loss (λ=0.1), the latter penalizing gradient mismatches at class boundaries between prediction and ground truth. Training and evaluation used Monte Carlo (repeated random subsampling) cross-validation with 80/20 subject-level splits. Resampling continued until every subject appeared in a validation set at least once; coverage was reached after 11 iterations (RS1–RS11), which were then used for ensemble prediction and refinement. During each fold, data augmentation was performed using the TorchIO library, including random flipping along the axial axis, affine transformations (rotation up to 30°, scaling ±5%), elastic deformations, Gaussian noise, motion artifacts, gamma adjustment, blurring, and ghosting. Each fold was trained for a fixed number of epochs with a batch size of 2 and optimized using Adam (learning rate 0.001). The number of training epochs (570) was selected based on convergence patterns observed in preliminary folds, ensuring stable Dice performance on the validation set without significant overfitting. Automatic mixed precision training (AMP) was used via PyTorch's GradScaler for efficiency. At the end of all folds, predictions from each were aggregated across all subjects and used as input to the second-stage refinement model. The refinement model was a lightweight 3D U-Net designed to correct boundary errors. It received two channels per sample—the normalized CISS image and the hard segmentation from the principal model—and produced a 6-class output. The network comprised three encoder blocks (32, 64, 128 channels; max pooling) and two decoder blocks with attention-gated skips; each block used two 3D convolutions with BatchNorm and ReLU, followed by a 1×1×1 classifier. (Loss: boundary-weighted Dice) Refinement was trained per fold using the principal model’s predictions. For inference, voxelwise ensembling across the 11 refined models used majority voting with a ≥ 0.5 agreement

threshold; voxels below threshold defaulted to background. Finally, we applied an automated post-processing pipeline to enforce anatomical consistency: small-cluster removal, side-aware dominance per slice, coronal hole filling, and continuity corrections across the optic nerve→chiasm→tract transition. Implementation used Python (PyTorch 1.13+), NiBabel for NIfTI I/O, and TorchIO for augmentation.

ON anatomical subdivisions

A.I. segmentation of the ON anatomical subdivisions yielded relatively more stable results at the level of the iOrb and iCran segments with respect to iCan. In particular, the iOrb mean DSC was 0.86 (SD ± 0.08; 95% CI 0.84–0.87) and the Jaccard index 0.76 (SD ± 0.10; 95% CI 0.73–0.78). As for the boundary agreement, HD95 was 1.75 (SD ± 2.96; 95% CI 1.10–2.41), HD was 4.09 (SD ± 3.64; 95% CI 3.29–4.90), and the ASSD was 0.46 (SD ± 0.68; 95% CI 0.31–0.61). Precision and recall were 0.88 (SD ± 0.06; 95% CI 0.87–0.89) and 0.85 (SD ± 0.11; 95% CI 0.82–0.87), respectively, while VS was 0.95 (SD ± 0.07; 95% CI 0.93–0.96).

Performance was lower for iCan, where the DSC was 0.71 (SD ± 0.09; 95% CI 0.69–0.73) and the Jaccard index 0.56 (SD ± 0.09; 95% CI 0.53–0.58). However, HD95 and HD were to 3.25 (SD ± 6.34; 95% CI 1.85–4.65) and 5.40 (SD ± 7.46; 95% CI 3.75–7.05), respectively, and the ASSD was 0.95 (SD ± 2.29; 95% CI 0.45–1.46). Precision and recall were 0.73 (SD ± 0.11; 95% CI 0.71–0.75) and 0.71 (SD ± 0.12; 95% CI 0.68–0.74). VS was 0.91 (SD ± 0.08; 95% CI 0.89–0.93). The lower DSC observed for the iCan segment contrasts with the relatively preserved volumetric similarity. This difference reflects the distinct sensitivity of the two metrics: DSC penalizes small boundary deviations, which are common in narrow structures such as the intracanalicular optic nerve, whereas VS mainly captures agreement in overall volume. In this context, the combination of reduced DSC and high VS indicates that most discrepancies arise

from local boundary uncertainty rather than from substantial over- or underestimation of the segment.

Results for iCran were similar to iOrb, with a DSC of 0.81 (SD ± 0.05; 95% CI 0.80–0.82) and Jaccard index of 0.68 (SD ± 0.07; 95% CI 0.67–0.70). HD95 and HD were 1.77 (SD ± 1.64; 95% CI 1.41–2.14) and 4.06 (SD ± 2.27; 95% CI 3.55–4.56) respectively, while the ASSD was 0.51 (SD ± 0.21; 95% CI 0.46–0.55). Precision and recall were 0.82 (SD ± 0.08; 95% CI 0.80–0.83) and 0.81 (SD ± 0.08; 95% CI 0.79–0.83), respectively, with VS of 0.94 (SD ± 0.05; 95% CI 0.92–0.95).

External single sample test.

To provide an illustrative example of applicability beyond the training domain, aVP-seg was run on a single high-resolution CISS dataset acquired at an external Center with a different scanner and acquisition protocol. (See Supplementary Figure 2) Although no quantitative evaluation was performed and no conclusions regarding generalizability can be drawn from a single case, the model successfully produced anatomically plausible segmentations of the optic nerves, chiasm, and optic tracts. Formal multi-center and multi-vendor validation remains necessary.

Supplementary Table S1. Summary of all spatial similarity metrics between Reader 1 (R1) and Reader 2 (R2) for the merged aVP ground-truth masks. Higher values of DSC, Jaccard, Precision, and Recall indicate greater volumetric overlap between raters. Lower values of HD, HD95, and ASSD reflect higher boundary similarity.

HD, HD95, and ASSD are expressed in millimeters (mm). All values are reported as mean, standard deviation (SD), and 95% confidence interval (95%CI). The CI for the mean assumes that sample means follow a *t* distribution with *N − 1* degrees of freedom.

Inter-reader analysis (R1vs R2)

		**95% Confidence Interval**		
	**Mean**	**Lower**	**Upper**	**SD**
**DSC**	0.83	0.82	0.84	0.03
**Jaccard**	0.71	0.70	0.72	0.05
**Precision**	0.85	0.84	0.86	0.05
**Recall**	0.82	0.81	0.83	0.05
**HD**	5.94	5.33	6.56	2.77
**HD95**	1.09	1.03	1.14	0.25
**ASSD**	0.44	0.42	0.46	0.10

Supplementary Table S2. Summary of all spatial similarity metrics between A.I. and GT, each aVP ON, OC and OT segment. Higher values for Dice, Jaccard, Precision, Recall, and Volumetric similarity, and lower values for Hausdorff, HD95, and Average surface distance, indicate higher morphology similarity. Hausdorff distance, 95th percentile Hausdorff distance (HD95), and average symmetric surface distance (ASSD) are measured in millimeters (mm). Volume-based metrics are expressed in cubic millimeters (mm^3), SD=standard deviation. 95% confidence interval= 95%CI.

Metric (95CI%)	ONL	ONR	ONC	OTL	OTR
**DSC**	0.85 (0.84–0.86), SD=0.05	0.86 (0.85–0.87), SD=0.04	0.83 (0.81–0.85), SD=0.09	0.74 (0.73–0.76), SD=0.07	0.75 (0.74–0.77), SD=0.07
**Jaccard**	0.75 (0.73–0.76), SD=0.07	0.76 (0.74–0.77), SD=0.07	0.72 (0.69–0.74), SD=0.11	0.60 (0.58–0.62), SD=0.09	0.60 (0.58–0.63), SD=0.11
**Precision**	0.87 (0.86–0.88), SD=0.05	0.87 (0.86–0.88), SD=0.05	0.90 (0.88–0.92), SD=0.09	0.76 (0.73–0.79), SD=0.14	0.73 (0.70–0.77), SD=0.15
**Recall**	0.85 (0.83–0.86), SD=0.08	0.85 (0.84–0.87), SD=0.07	0.79 (0.76–0.82), SD=0.13	0.76 (0.73–0.79), SD=0.13	0.79 (0.76–0.82), SD=0.14
**Volumetric Similarity**	0.96 (0.95–0.97), SD=0.04	0.96 (0.95–0.97), SD=0.04	0.89 (0.87–0.91), SD=0.09	0.88 (0.86–0.9), SD=0.10	0.88 (0.86–0.90), SD=0.09
**HD**	3.17 (2.68–3.66), SD=2.21	3.03 (2.49–3.56), SD=2.43	2.22 (1.99–2.44), SD=1.01	4.76 (4.09–5.42), SD=3.013	4.57 (4.10–5.03), SD=2.10
**HD95**	1.32 (1.00–1.64), SD=1.44	1.42 (1.06–1.79), SD=1.64	1.47 (1.29–1.66), SD=0.84	2.33 (2.00–2.66), SD=1.49	2.28 (2.00–2.57), SD=1.30
**ASSD**	0.39 (0.34–0.44), SD=0.21	0.39 (0.34–0.44), SD=0.24	0.46 (0.40–0.51), SD=0.24	0.65 (0.59–0.71), SD=0.27	0.65 (0.59–0.71), SD=0.26

Supplementary Figures

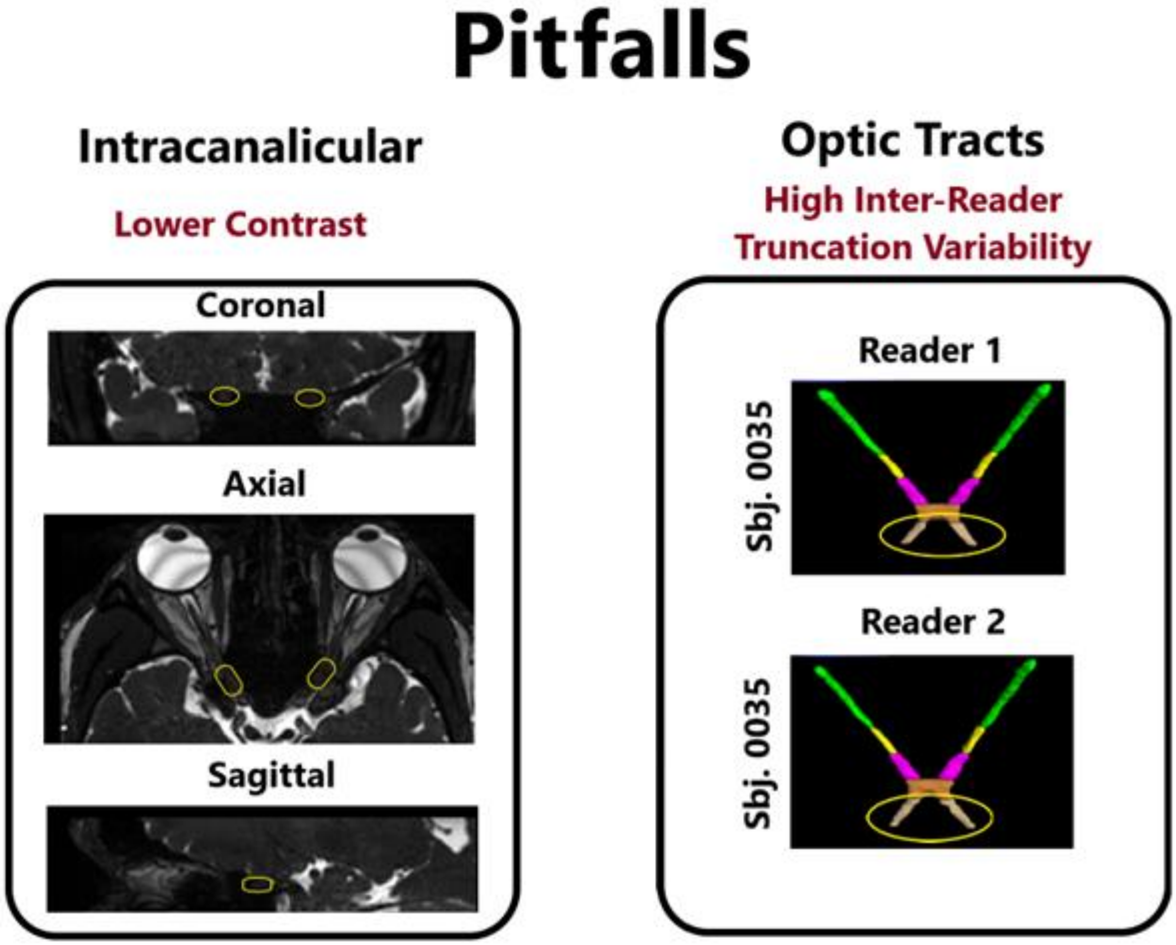

Supplementary Figure 1. Common anatomical pitfalls in the segmentation of the aVP.

Left: *Intracanalicular segment* CISS images (coronal, axial, sagittal) showing reduced contrast between the optic nerve and surrounding cerebrospinal fluid, leading to boundary uncertainty.

Right: *Optic tracts* example from subject 0035 illustrating high inter-reader variability in defining the posterior truncation of the tract. Yellow ellipses indicate regions of disagreement between readers.

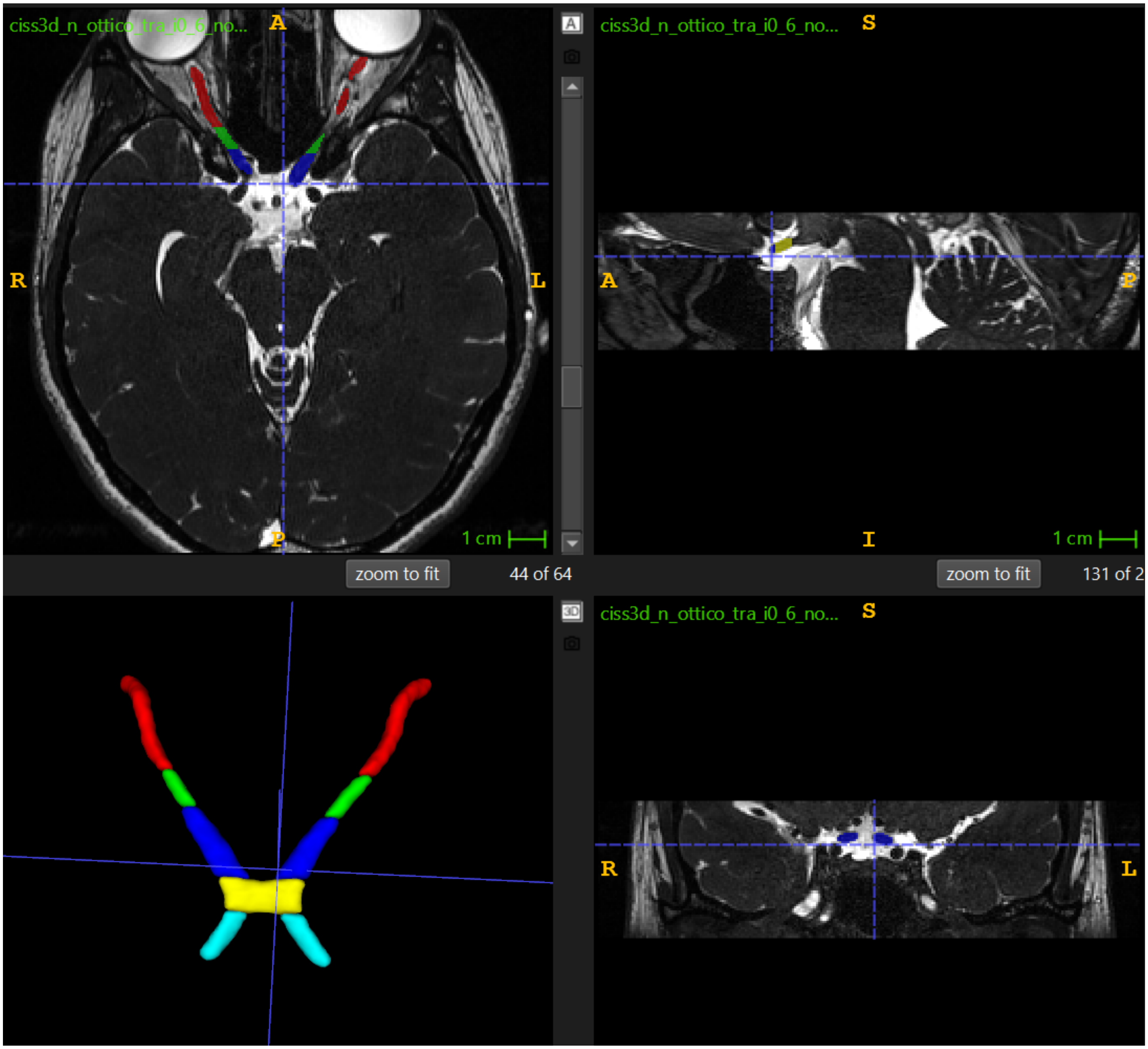

Supplementary Figure 2. Representative axial, coronal, and sagittal CISS images from an external subject acquired at a different center using a different scanner with the same acquisition protocol are shown, with the automated aVP-seg output overlaid. The segmentation delineates the optic nerves, chiasm, and optic tracts using color coding, together with the corresponding 3D volume rendering. This example is provided for illustrative purposes only; no quantitative evaluation was performed, and no conclusions regarding cross-scanner or cross-protocol generalizability can be drawn from a single case.
